# Emergence of diversity in carbapenemase-producing *Escherichia coli* ST131, England, January 2014 to June 2016

**DOI:** 10.2807/1560-7917.ES.2019.24.37.1800627

**Published:** 2019-09-12

**Authors:** Nicholas Ellaby, Michel Doumith, Katie L Hopkins, Neil Woodford, Matthew J Ellington

**Affiliations:** 1 Antimicrobial Resistance and Healthcare Associated Infections (AMRHAI) Reference Unit, National Infection Service, Public Health England, London, United Kingdom; 2 Infectious Diseases Research Department, King Abdullah International Medical Research Center, Riyadh, Saudi Arabia; 3 King Saud bin Abdulaziz University for Health Sciences, Riyadh, Saudi Arabia

**Keywords:** XDR, resistance spread, evolving resistance, horizontal transfer, United Kingdom, healthcare-associated infections, bacterial infections, Escherichia coli, antimicrobial resistance, laboratory surveillance, epidemiology

## Abstract

**Background:**

*Escherichia coli* ST131, a global, high-risk clone, comprises fluoroquinolone resistance (FQ-R) mutations and CTX-M extended-spectrum beta-lactamases associated with the *fimH*30-encoding clades, C1 and C2. Further carbapenem resistance development in ST131 is a public health concern.

**Aim:**

This observational study aimed to probe the diversity of carbapenemase-producing *E. coli* (CP *E. coli*) ST131 across England.

**Methods:**

ST131 isolates were identified using whole-genome sequencing (WGS) data generated for all non-duplicate CP *E. coli* from human samples submitted to the national reference laboratory from January 2014 to June 2016. Antimicrobial resistance (AMR) gene content and single nucleotide polymorphism (SNP) data were compared against a published ST131 phylogeny and analysed alongside patient metadata.

**Results:**

Thirty-nine genetically diverse ST131 CP *E. coli*, from eight of nine regions, represented 10% of CP *E. coli* isolates sequenced. Ten and eight isolates were from the FQ-susceptible (FQ-S) clades A and B, while eight and 15 isolates belonged to the FQ-R clades C1 or C2, respectively. Seven distinct carbapenemases were identified: KPC-2 (21 isolates, 6 regions) frequently occurred among clade C2 isolates (n = 10). OXA-48-producers (10 isolates, 3 regions) were often from clade A (n = 5). NDM-1 (n = 4), NDM-5 (n = 1), VIM-1 (n = 1), VIM-4 (n = 1) and OXA-181 (n = 1) were also identified. Clade C2 isolates encoded more AMR genes than those from clades A (p = 0.02), B (p = 9.6 x 10^−3^) or C1 (p = 0.03).

**Conclusion:**

When compared with its global predominance among ESBL-*E. coli,* ST131 represented a fraction of the CP *E. coli* received, belonging to diverse clades and encoding diverse carbapenemases. The greater accumulation of resistance genes in clade C2 isolates highlights the need for ongoing monitoring of this high-risk lineage.

## Introduction

The increasing incidence of antimicrobial resistance worldwide and a paucity of new drugs in development presents a major threat to the treatment of bacterial infections [1,2]. Since the millennium, the successive emergences of *Escherichia coli* with horizontally acquired CTX-M extended-spectrum beta-lactamases (ESBLs) and, more recently, carbapenemases, have heralded the advent of resistance to last-resort treatment options for many serious infections because of *E. coli* and other Enterobacterales.

The pandemic of resistant *E. coli* has been associated particularly with the uropathogenic *E. coli* lineage ST131. The population structure of the ST131 lineage was shaped by the acquisition of virulence and resistance elements which has resulted in three dominant clades; A, B and C [1,2]. Around 1980, clade B diversified into clade C via subclades B0 and C0. Clade C strains were characterised by distinct alleles for the genes *gyrA* (coding for gyrase) and *parC* (coding for topoisomerase) which impart elevated fluoroquinolone resistance (FQ-R) [1,2]. This clade was further sub-divided according to *fimH* gene variants into the C1 (*fimH30*-R) and C2 (*fimH30*-Rx) clades in 1987 [2,3]. The gene *fimH* encodes for the type 1 fimbrial adhesin which binds to, and facilitates colonisation of, the bladder epithelium [4,5] and has been suggested as an epidemicity factor for ST131. The C2 clade also became the principle clone associated with the spread of *E. coli* carrying CTX-M-15 ESBL [6] and multiple other resistance genes [7].

Recent evidence has demonstrated ongoing dynamics and diversification of clade C2, with the emergence and spread of new resistant forms. An example of further acquisition and proliferation in the ST131 clades has been observed with CTX-M-27 ESBL-encoding isolates being reported first in Japan, followed by India and then northern Europe [8-10]. Concerns over the continued acquisition and expansion of the ST131 resistance repertoire were manifested by early reports of the NDM carbapenemase occurring in India [11] and Vietnam [12], as well as scattered reports of the *kpc* gene occurring in ST131 globally [13]. Other carbapenemases such as VIM, IMP and OXA-48-like enzymes have been reported to a lesser extent [14,15]. Importantly, CP *E. coli* have also recently been recorded in Spanish rivers [16] and long-term care facilities in Italy [17]. This observation of carbapenemase-expressing CP *E. coli* globally across many environmental niches demonstrates the breadth of reservoirs that contain CP *E. coli*.

In the United Kingdom (UK), ST131 has been widely described in *E. coli* from community- and hospital-onset infections alike [18]. However, there has been a relative paucity of carbapenemase-producing ST131 isolates investigated to date. This observational study augments an existing ST131 phylogeny with clinical isolates encoding carbapenemases from across England, and it evaluates the genetic, geographical and temporal diversity present among the ST131 carbapenemase-producing *E. coli* (CP *E. coli*) in England.

## Methods

### Bacterial isolates and whole genome sequencing

CP *E. coli* were isolated from human samples by clinical diagnostic laboratories and submitted to Public Health England’s (PHE) Antimicrobial Resistance and Health Care Associated Infections (AMRHAI) Reference Unit for carbapenemase gene detection and/or antimicrobial susceptibility testing. The referring laboratories were located in the nine English Regions (London, East of England, South East, South West, West Midlands, North West, North East, Yorkshire and the Humber and East Midlands). Between January 2014 and June 2016, AMRHAI undertook whole genome sequencing (WGS) of every initial CP *E. coli* isolate received per patient. DNA was extracted from RNase-treated lysates via a QIAsymphony DSP DNA Midi Kit (Qiagen GmbH, Hilden, Germany). DNA libraries were prepared using the Nextera XT sample preparation method and sequenced with a standard 2 x 101 base protocol on a HiSeq 2500 (Illumina, San Diego, California, United States (US)).

Data for carbapenemase-producing ST131 *E. coli* from different patients were identified and extracted for analysis.

Ethical approval was not required as no personal identifiable information was used in this study. Short-read sequence data for the 39 CP *E. coli* ST131 isolates were submitted to the European Bioinformatic Institute’s European Nucleotide Archive as project PRJEB32306.

### Antimicrobial susceptibilities

Minimum inhibitory concentrations (MICs) of polymyxins, beta-lactams, aminoglycosides, carbapenems, tetracyclines and fluoroquinolones were determined at the time of receipt (January 2014– June 2016) by BSAC agar dilution methodology and were interpreted according to the European Committee on Antimicrobial Susceptibility Testing (EUCAST) guidelines version N.0, 2018) for 26 of the CP *E. coli* samples.

### Whole genome sequencing data analysis

WGS data were deplexed via Casava, and nucleotides with a Phred score less than Q30 at the ends of the reads were removed with Trimmomatic [17]. Species ID was determined with Kmer-ID [19]. MLST profile was determined by the mapping tool MOST [20]. The in house tool GeneFinder [21] mapped sequenced reads to reference sequences with bowtie2 and generated an mpileup file with SAMtools version 0.1.18 [22] which enabled the rapid detection of antimicrobial resistance gene complements and plasmid replicon types [18,20].

To provide a contextual framework for the 39 ST131 CP *E. coli* isolates from England, additional raw read sequences for 188 global non-carbapenemase producing ST131 isolates identified and validated previously [1] were retrieved from the National Center for Biotechnology Information (NCBI) Short Read Archive along with the EC958 assembled reference genome. Raw reads were analysed with PRINSEQ version 0.20.4 [23] and trimmed with a mean base quality score of ≥ 30 and a read length ≥ 70% of the expected read length.

Single nucleotide polymorphisms (SNPs) were determined through read mapping using the PHEnix pipeline [24]. Briefly, the quality filtered reads were mapped against reference sequence EC958 using bwa_mem, followed by variant detection via Genome Analysis Toolkit (GATK) with a minimum depth of 5, mapping quality (MQ) score of 30, allele depth ratio of 0.90, quality score of 40 and an MQ ratio of 0.1. Using methods and parameters described by Stoesser et al. [2], the variant call format (VCF) files base calls were retained if: (i) the percentage of high quality base calls was ≥ 90% and ≥ 5 high-quality bases were observed; (ii) the root of the mean square mapping quality of reads covering the putative variable site was ≥ 30; (iii) Phred scaled quality of a base call was ≥ 25; and (iv) reads spanning variable sites had high quality bases that made up ≥ 35% of variable sites [2]. Suspected recombinant regions were removed using Gubbins [25] and phylogenetic trees were constructed with RAxML version 8 using the general time-reversible Gamma model of among-site rate variation, and validated using 1,000 bootstrap repetitions to assess nodal support [26]. All trees were then viewed within the R statistics package with the ggtree library [27].

### Bayesian temporal and geographical analysis

TempEST was used to assess the variance between the time of sampling and the root-to-tip divergence in maximum likelihood trees [28] using heuristic residual mean squared function (residual mean square = 6.45 x 10^−6^; correlation coefficient = 0.5059; R^2^ = 0.256). BEAST 2 version 2.4.8 [29] was used to perform Bayesian temporal analysis on maximum likelihood trees to reduce variance and investigate cladal expansion for ST131 CP *E. coli* using the 3,779-bp non-recombinant SNPs conserved across all 227 ST131 isolates. As only SNP sites were used in the alignment a Gamma site model (Gamma Category count 0) was used, which assumes a gamma distribution for site-to-site rate heterogeneity, and the proportion of invariant sites was fixed at 0%. The Tamura-Nei 1993 (TN93) model, which weights transitions and transversion mutations according to their likelihood, was found to be the best model to represent the phylogeny (path sampling maximum likelihood estimates are described in Supplementary Table S1A and effective sample size scores (ESS) in Supplementary Table S1B). A strict molecular clock, constant population size and uniform clock rate was found to be the most appropriate model as all isolates were from the same *E. coli* sequence type. The suitability of this model was reflected in the ESS scores when compared against GTR, HKY and JC69 site models under like-for-like parameters. To ensure convergence, Markov Chain Monte Carlo (MCMC) generations for each analysis were performed in triplicate for 30 million steps (totalling 90 million iterations) sampling every 1,000 steps, producing ESS scores equal to or greater than 200 (Supplementary Table S1B). Replicate analyses were then combined using the BEAST program LogCombiner with 10% burn-in.

T-test, X^2 ^and ANOVA statistical tests were all performed using standard libraries contained within the R statistics package [30].

## Results

### Diverse carbapenemase-producing *Escherichia coli* ST131 in England

Thirty-nine ST131 CP *E. coli* isolates were referred to and sequenced at PHE’s AMRHAI reference unit between January 2014 to June 2016 (30 months) from 20 laboratories that represented eight of the nine English regions. These accounted for 10% of the total number of CP *E. coli* isolates submitted. Temporal analysis showed that the average number of CP *E. coli* ST131 isolates submitted to PHE on a monthly basis rose from 0.67 in 2014 (ST131 CP *E. coli* = 8), to 1.67 in 2015 (ST131 CP *E. coli* = 20), and to 1.83 in 2016 (first 6 months only, ST131 CP *E. coli* = 11). As a percentage of the total CP *E. coli* isolates across England, ST131 represented 4.5% in 2014 (CP *E. coli* = 177), 6.2% in 2015 (CP *E. coli* = 324) and 4.9% in the first half of 2016 (CP *E. coli* = 223). This increase in CP *E. coli* was linked to the number of KPC-2 producing isolates (mean/month: 2014 = 0.33; 2015 = 0.92; 2016 = 1.00) and OXA-48 producing isolates (mean/month: 2014 = 0.08; 2015 = 0.42; 2016 = 0.67).

**Figure 1:**
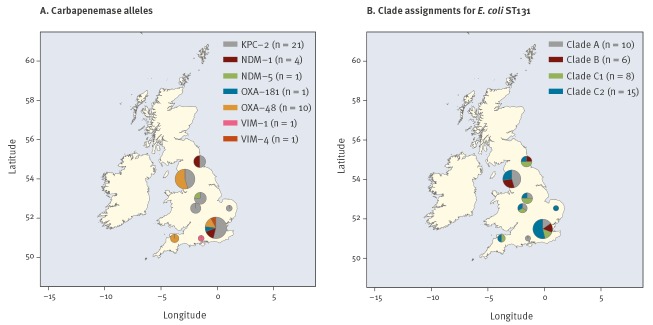
Geographical distribution of carbapenemase-producing *Escherichia coli* ST131 isolates, England, January 2014–June 2016 (n = 39)

Analysis of all PHE CP *E. coli* ST131 isolates in this study for the occurrence of regional or local clusters, irrespective of time, showed that the majority were isolated from London (n = 13) or the North West region (n = 11) (Figure 1A). Seven distinct carbapenemase alleles were detected, but only the KPC-2, OXA-48 and NDM-1 carbapenemases were encoded by isolates from multiple regions. Of these, KPC-2 was the most numerous (n = 21) and widely distributed (isolated in 6 regions), with the largest number of isolates identified in London (n = 7). The second most frequent allele, OXA-48, occurred in 10 ST131 CP *E. coli* and was found most often in the North West region (n = 6). The remaining carbapenemase alleles occurred sporadically across London (NDM-1, OXA-181 and VIM-4), the North East (NDM-1), West Midlands (NDM-5) and the South East (VIM-1). None of the isolates in this study were associated with known outbreaks of CP *E. coli*. (Figure 1A, Supplementary Table S2).

Pie charts were scaled according to the number of isolates.

Comparison of WGS data from the CP *E. coli* ST131 with a previously published WGS-based population structure for ST131 identified that 23 of 39 ST131 CP *E. coli* from across England were from the FQ-R clades C1 (n = 8) and C2 (n = 15). Clade C2 was the most widely distributed clade of ST131 CP *E. coli* (isolated from 7 regions) and was the most highly represented clade in three regions (Figure 1B). The FQ-S clades, A and B, were relatively well represented (10 and 6 isolates, respectively) and widely disseminated (found in 5 and 3 regions, respectively). Opposite cladal biases were apparent in the two regions with the largest numbers of isolates: in London (n = 13), clades C1 and C2 predominated (2 and 7 isolates, respectively); whereas clades A and B predominated in the North West region (5 and 3, respectively) (Figure 1B, Supplementary Table S2).

The combined data for ST131 clades and carbapenemase alleles indicated that isolates from the largest CP *E. coli* clade, C2, were most frequently associated with KPC-2 (n = 13) and predominated among ST131 CP *E. coli* isolated in London (n = 7). In contrast, the second most frequent carbapenemase, OXA-48 (n = 10), occurred most often in clade A isolates from the North West region (n = 5), demonstrating the contrasting phylogeographical patterns in ST131 CP *E. coli* for the two most frequent carbapenemases.

### Emergence and expansion of clade C2

**Figure 2:**
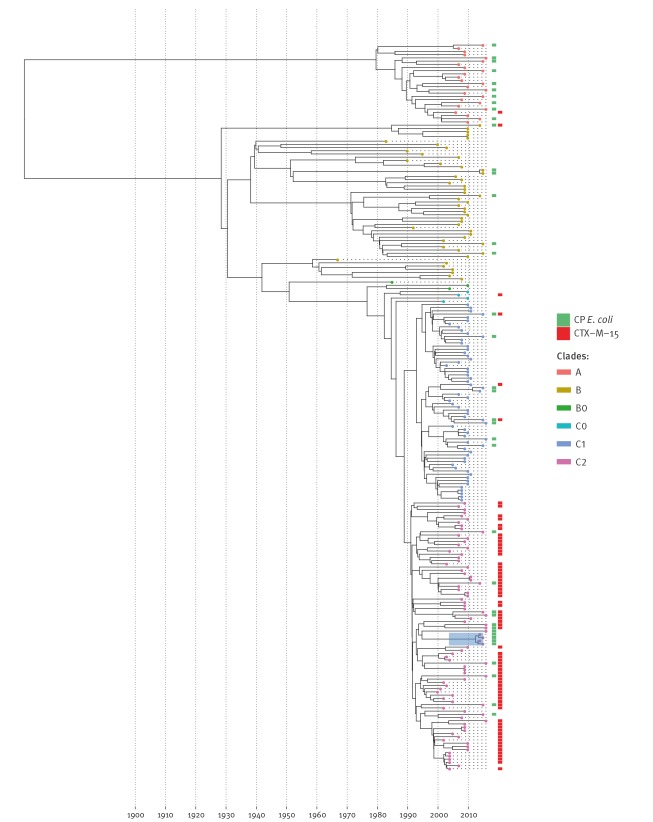
Evolutionary phylogeny of carbapenemase-producing *Escherichia coli* from England within the larger, global ST131 phylogeny previously validated by Stoesser et al. [2] and Ben Zakour et al. [1]

To assess the extent of any clonality and clade specific expansion, the CP *E. coli* isolates were incorporated into and visualised within the context of a previously validated phylogenetic BEAST tree [1]. The CP *E. coli* isolates were widely distributed across the diversity present in each of the clades with little evidence for clear expansions of sub-clades or genetic clusters of carbapenemase producers emerging, although there was a close relative cluster in clade C2 (Figure 2). This group represented KPC-2 isolates without a CTX-M-15 gene from London, two isolates from 2014 and two from 2015, and the North West region, one isolate from 2016.

The blue box indicates a newly emerged KPC-2, CTX-M-15 negative lineage within clade C2.

MIC analyses for 26 of 39 isolates highlighted 12 isolates of ST131 CP *E. coli* that were phenotypically resistant to the greatest number of antimicrobials, representing at least seven drug classes (carbapenems, other beta-lactams, including third generation cephalosporins, fluoroquinolones, trimethoprim, tetracyclines, sulfonamides and aminoglycosides). Those 12 most resistant isolates remained susceptible to colistin and tigecycline. They originated from seven English regions, but were most often isolated in London (n = 6). We observed that 10 of the 12 isolates were from clade C2 and originated from six regions. Seven of the 10 isolates from clade 2 encoded KPC-2, two encoded OXA-48, and the remaining isolate encoded VIM-4 (Supplementary Table S3).

**Figure 3:**
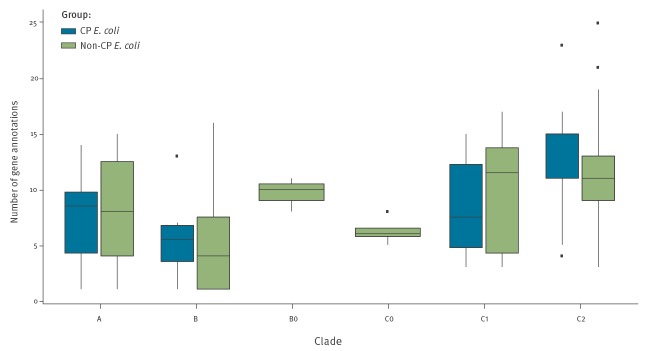
Antimicrobial resistance genes per *Escherichia coli* isolate (CP *E.coli*, England, 2014–2016: n = 39; non-CP *E. coli*: n = 188)

To better explore the suggestion that isolates from the C2 clade had a propensity to have a greater number of antimicrobial resistances, we examined the number of AMR genes per isolate. The number of AMR genes did not differ significantly between isolates expressing different carbapenemase genes (ANOVA: p = 0.826), but AMR gene counts were affected by the clade that an isolate belonged to (ANOVA: p = 0.0214). Clade C2 isolates were associated with significantly higher numbers of acquired AMR genes (mean = 11.1; median = 13.0) (Figure 3) (t-test: clade A, p = 0.02, mean = 8.1, median = 8.5; clade B, p = 9.6 x 10^−3^, mean = 6.3, median = 5.0; clade C1, p = 0.03, mean = 8.25, median = 6.5). This was attributable to mobile genes that encoded resistance to aminoglycosides (X^2^ = 16.657; df = 6; p = 0.01); fluoroquinolones (X^2^ = 9.958; df = 3; p = 0.02) and sulfonamides (X^2^ = 9.9789; df = 2; p = 6.8 x 10^−3^). Further comparisons revealed that clade C2 CP *E. coli* also had more AMR genes than their non-CP *E. coli* C2 counterparts (X^2^ = 35.177; df = 19; p = 0.01), despite almost half (7/15) lacking the CTX-M-15 gene that has previously been strongly associated with the multi-resistant status of clade C2. Supplementary Table S4 describes the most common resistance genes in CP *E. coli* and non-CP *E. coli* isolates.

CP: carbapenemase-producing.

Isolates groupings are shown according to clade and separated into carbapenemase-encoding and non-carbapenemase-encoding sub-groups. Only one bar is seen for Clade B0 and C0 because no carbapenemase isolates submitted to Public Health England were identified as such. Dots represent outliers.

### Plasmid replicons

The plasmid replicon sequences found in the 39 CP *E. coli* ST131 isolates most often indicated the presence of replicons from the FIA (n = 23), FII (n = 35) and FIB (n = 35) plasmid incompatibility groups (Supplementary Table S5). Non F-type replicons were less evenly distributed across clades as indicated by the higher numbers in isolates from clades A (mean = 2.2) and C2 (mean = 2.13) compared with isolates in clades B (mean = 0.67) and C1 (mean = 0.88) (ANOVA, p = 5.59 x 10^−5^). For isolates in clade A, particular carbapenemase types did show unique associations with replicons, with VIM-1/-4 associated with A/C replicons, KPC-2 to N-type replicons and OXA-48 association with IncL/M replicons. Isolates in the other clades (B, C1 and C2) showed no clear relationships between unique plasmid replicons and particular carbapenemase genes (Supplementary Table S5).

## Discussion

The observation that ST131 CP *E. coli* represented only 10% of CP *E. coli* isolates that were submitted to the reference laboratory over the study period contrasted the current narrative that ST131 shows a global predominance among ESBL-producing *E. coli*. Nevertheless, the wide distribution of ST131 CP *E. coli* across all but one of the nine English regions indicates the ongoing success of ST131 *E. coli* and highlights its progression beyond being an ESBL encoding lineage in England. The diversity of clades and carbapenemase alleles also greatly differed from the emergence of a single lineage akin to the CTX-M-15 expressing C2 isolates responsible for the pandemic established in the mid-2000s. While no evidence was found for distinct newly emerged phylogenetic clusters reaching predominance, the relative success of clade C2 in particular highlights the potential to repeat the clonal success of the CTX-M-15 positive clade [1]. Specifically, the five KPC-2 encoding isolates with relative relatedness spanning 3 years and two locations indicate the potential for expansion within clade C2. Moreover, the phylogeographic diversity of isolates from C1 and C2 clades demonstrates the success of diverse isolates from within these clades as carbapenemase producers, and highlights the importance of ongoing monitoring in order to help to identify any further expansion of such isolates.

The contrast between the two most successful carbapenemases were marked. The predominance of KPC-2 among the FQ-R C2 isolates from London occurred contemporaneously to a large outbreak of KPC-2 in multiple species of the Enterobacterales in the North West region [31]. In that context, the predominance of OXA-48 among mostly FQ-S clade A isolates in the North West region did not coincide with the main clinical, healthcare-associated, circulation of CP *E. coli* in the region at the time. The principal mechanism of antibiotic gene acquisition in bacteria is through horizontal gene transfer, which is primarily mediated by plasmids. It is not possible to ascertain from short-read data whether these OXA-48 positive clade A isolates were part of a wider circulation of isolates and/or plasmids in the area at the time. However, nine of 10 OXA-48-positive isolates had reads mapping to the origin of replication for IncL/M-type plasmids, which have previously been identified as the main vehicles encoding OXA-48 in many Enterobacterales species [32-36]. For KPC-2, potential plasmid vectors appeared to be more diverse and highlighted the mobility that underpins the spread of carbapenemase genes. However, accurately attributing plasmids to genes was limited by the use of short-read data in this project. Ongoing studies using additional long-read sequencing data will be required to better determine, monitor and survey the extent of mobile element and AMR flux in important, potentially public health relevant clones such as ST131.

The marked differences in the multi-resistance gene profiles, evidenced by the higher number of AMR genes noted in clade C2 when compared against their ESBL-positive counterparts, suggests that clade C2 may have been differentially affected, as compared with clades A-C1, over time by a pressure to accumulate multiple resistances. This may be because of clade C2 isolates being exposed more regularly to antimicrobial pressure in healthcare settings. We speculate that clade C2 isolates possess a mechanism(s) or are otherwise predisposed towards the increased acquisition of resistance genes; and we are investigating this further. The detection of repeated accumulation of resistance genes in this epidemic *E. coli* clade is concerning. The example of *Klebsiella pneumoniae* has shown that some high-risk clones spread in successive forms, first encoding ESBLs (CTX-M-15), followed by carbapenemases and subsequently developing resistance to colistin (an agent of last resort) [37]. This progression of resistance accumulation presents significant challenges in infection control, particularly within clinical settings. The level of concern for such a pattern in *E. coli* and especially ST131 would be further heightened by the emergence and wide geographic dissemination of mobile colistin resistance (*mcr*) genes in the species, not least as these have already been reported on IncHI2-based multi-resistance plasmids that are mobile and can replicate in *E. coli* and other members of the Enterobacterales [21].

The data presented highlight the ongoing diversification and success of the clade C ST131 CP *E. coli* within England, the emergence of multiple new resistance profiles across ST131 and a propensity for these to be newly-emerging, increasingly-resistant forms of clade C2 in particular. Detecting genomic signals associated with the repeated acquisition and evolution of resistance profiles in the most successful *E. coli* clades provides a platform on which to base healthcare strategies and interventions that can assist in the early detection and mitigation of high-risk lineages in the future. It also underlines the importance of continued monitoring of this high-risk lineage with known pandemic potential, given the current lack of good alternatives to carbapenems for the treatment of Gram-negative antimicrobial-resistant infections.

## Supplementary Data

3476000 Supplementary Material
