# Supplementary material for: Emergence of diversity in carbapenemase-producing Escherichia coli ST131, England, January 2014 to June 2016
Source: Euro Surveill. 2019 Sep 12;24(37):1800627. doi: 10.2807/1560-7917.ES.2019.24.37.1800627 (PMC6749775; doi:10.2807/1560-7917.ES.2019.24.37.1800627)
Supplement: Supplementary Material [file 1800627_ELLINGTON_SupplementaryMaterial.pdf]

Supplementary Material

This supplementary material is hosted by *Eurosurveillance* as supporting information alongside the article “Emergence of diversity in carbapenemase-producing Escherichia coli ST131, England, January 2014–June 2016.” on behalf of the authors who remain responsible for the accuracy and appropriateness of the content. The same standards for ethics, copyright, attributions and permissions as for the article apply. *Eurosurveillance* is not responsible for the maintenance of any links or email addresses provided therein.

Supplementary Table S1A Path sampler results for TN93, GTR, HKY and JC69 model comparisons using the following parameters; alpha 0.3, steps = 4, chain length = 100,000, burn in = 50%, pre-burnin = 100,000.

		TN93			GTR		
Step	theta	likelihood	contribution	ESS	likelihood	contribution	ESS
0	1.00	-75931.99	-30558.08	6.00	-74876.95	-30210.35	3.21
1	0.60	-75391.30	-20551.31	4.64	-74788.97	-20383.93	8.99
2	0.33	-75550.69	-12930.76	3.38	-74918.04	-12815.62	9.63
3	0.15	-76161.97	-7291.65	7.65	-75633.16	-7237.21	16.99
4	0.06	-79252.25	-3515.64	5.13	-78301.41	-3470.03	13.58
5	0.02	-94351.27	-1322.20	12.63	-93524.34	-1313.43	7.17
6	0.00	-165586.64	-260.63	16.96	-167839.58	-269.28	11.31
7	0.00	-2764310.68	0.00	6.99	-2424104.69	0.00	6.65
marginal L		-76430.28			-75699.85		

		HKY			JC69		
Step	theta	likelihood	contribution	ESS	likelihood	contribution	ESS
0	1.00	-75820.02	-30618.23	2.89	-78610.32	-31650.82	2.89
1	0.60	-75303.39	-20527.57	7.29	-78064.27	-21279.41	4.17
2	0.33	-75538.15	-12922.69	15.85	-78328.26	-13402.41	4.12
3	0.15	-76213.03	-7295.42	7.17	-78901.47	-7555.21	6.72
4	0.06	-79429.20	-3523.16	4.58	-81396.66	-3597.59	16.98
5	0.02	-93766.34	-1315.57	4.47	-97888.86	-1371.35	13.17
6	0.00	-170346.95	-278.74	4.98	-169439.19	-269.06	4.18
7	0.00	-2865552.56	0.00	13.07	-2407681.39	0.00	501.00
marginal L		-76481.38			-79125.84		

Supplementary Table S1B Summary of effective sample size scores generated from BEAST reconstructed phylogenies for the 39 CP *E. coli* and 188 non-CP *E. coli.*

Statistic	GTR		TN93		HKY		JC69	
	Mean	ESS	Mean	ESS	Mean	ESS	Mean	ESS
posterior	-76253.87	286	-76820.30	523	-76835.50	25	-79479.35	21
likelihood	-74493.52	154	-75070.24	284	-75090.81	24	-77735.83	20
prior	-1760.34	227	-1750.06	667	-1744.69	473	-1743.53	260
treeLikelihood	-74493.52	154	-75070.24	284	-75090.81	24	-77735.83	20
TreeHeight	152.89	1196	152.58	1097	151.15	720	150.75	444
clockRate	0.00	323	0.00	639	0.00	432	0.00	269
popSize	802.42	2219	805.66	1916	796.96	1417	802.65	865
CoalescentConstant	-1736.87	721	-1737.65	668	-1735.15	473	-1736.84	260
rateAC	0.60	37	NA	NA	NA	NA	NA	NA
rateAG	2.06	36	NA	NA	NA	NA	NA	NA
rateAT	0.71	37	NA	NA	NA	NA	NA	NA
rateCG	0.11	39	NA	NA	NA	NA	NA	NA
rateGT	0.60	37	NA	NA	NA	NA	NA	NA
rateCT	2.06	36	NA	NA	NA	NA	NA	NA
freqParameter.1	0.20	3251	0.21	2635	0.21	5217	NA	NA
freqParameter.2	0.30	3361	0.28	3408	0.28	4869	NA	NA
freqParameter.3	0.30	3757	0.29	3798	0.29	4622	NA	NA
freqParameter.4	0.20	3486	0.22	3133	0.22	5232	NA	NA
kappa	NA	NA	4.97	3626	4.98	7346	NA	NA
kappa2	NA	NA	4.98	3494	NA	NA	NA	NA

Supplementary Table S2: The breakdown of clade-carbapenemase ST131 CP *E. coli* isolate groups according to geographical location.

Region	Clade	Allele	No. of Isolates
East Anglia (N=1)	C2	KPC-2	1
London (N=13)	A	KPC-2	1
		VIM-4	1
	B	KPC-2	2
	C1	NDM-1	1
		OXA-48	1
	C2	KPC-2	4
		NDM-1	1
		OXA-181	1
		OXA-48	1
Midlands (N=4)	A	KPC-2	1
	C1	KPC-2	1
		NDM-5	1
	C2	KPC-2	1
North East (N=4)	B	NDM-1	1
	C1	KPC-2	2
	C2	NDM-1	1
North West (N=11)	A	OXA-48	5
	B	KPC-2	2
		OXA-48	1
	C2	KPC-2	3
South (N=1)	A	VIM-1	1
South West (N=2)	C1	OXA-48	1
	C2	OXA-48	1
West Midlands (N=3)	A	KPC-2	1
	C1	KPC-2	1
	C2	KPC-2	1

Supplementary Table S3. MIC results for CP *E. coli* isolates.

CP *E. coli* Isolate	Carbapenemase Allele	Clade	Amikacin	Ampicillin	Amoxicillin / Clavulanate	Aztreonam	Ceftazidime	Ceftazidime / Clav-ESBL	Ciprofloxacin	Cefotaxime / cloxacillin	Colistin	Cefepime	Cefepime / Clav-ESBL	Cefotaxime	Cefotaxime/ Clav-ESBL	Ertapenem	Cefoxitin	Gentamicin	Imipenem	Imipenem / EDTA-MBL	Meropenem	Minocycline	Piperacillin	Piperacillin / Tazobactam	Temocillin	Tigecycline	Tobramycin
1	KPC-2	A	4	>64	>64	64	16	16	0.13	8	≤0.5	8	2	8	4	16	16	0.5	8	4	8	16	>64	>64	16	0.25	0.5
2	KPC-2	C1	2	>32	64	16	4	4	>8	2	≤0.5	2	1	4	2	4	8	0.5	2	1	2	2	>64	64	16	0.25	0.5
4	OXA-48	B	2	>64	>64	≤0.13	0.25	0.25	0.13	1	≤0.5	0.25	0.13	1	0.5	1	8	0.25	1	0.5	0.25	2	>64	>64	>128	0.25	0.5
5	OXA-48	C1	2	>64	>64	16	16	1	>8	128	≤0.5	16	0.5	256	8	4	16	0.5	2	1	1	nt	nt	>64	>128	0.25	1
6	OXA-48	A	2	>64	>64	32	8	4	0.25	4	≤0.5	4	2	16	2	8	16	>32	8	4	4	nt	nt	>64	16	0.25	8
8	OXA-48	C2	8	>64	>64	16	4	4	>8	2	≤0.5	2	1	4	2	8	8	1	4	2	2	2	nt	>64	8	0.5	>32
9	VIM-1	C1	4	>64	>64	32	4	4	>8	2	≤0.5	2	1	32	2	8	32	1	4	4	4	nt	nt	64	8	0.25	1
10	VIM-4	C2	2	>64	>64	64	64	2	>8	256	≤0.5	32	1	>256	8	4	16	16	1	0.5	0.5	nt	nt	>64	>128	0.25	16
11	KPC-2	B	2	>64	64	0.5	>256	>32	0.13	256	≤0.5	64	>32	256	>32	>16	>64	32	32	0.25	>32	2	>64	>64	64	0.25	4
12	KPC-2	C2	2	>64	>64	64	8	4	>8	4	≤0.5	4	2	4	2	8	16	0.5	4	2	2	1	nt	>64	8	0.25	16
13	KPC-2	A	2	>64	>64	≤0.13	0.25	0.25	0.25	1	≤0.5	0.25	0.25	2	0.5	4	16	32	1	0.5	0.5	1	nt	>64	>128	0.25	4
14	KPC-2	C1	2	>64	>64	32	4	2	>8	2	≤0.5	2	2	8	2	8	8	0.5	4	2	2	2	nt	>64	8	0.25	1
15	NDM-1	C2	2	>64	>64	>64	64	2	>8	256	≤0.5	32	2	>256	1	4	16	32	4	2	2	1	nt	64	32	0.25	8
16	OXA-48	C1	2	>64	>64	16	4	2	>8	4	1	4	4	8	2	4	16	1	8	8	4	1	>64	>64	8	0.25	1
17	KPC-2	C2	>64	>64	>64	>64	256	>32	>8	256	1	64	4	>256	>32	16	>64	>32	8	4	4	2	nt	>64	32	0.25	16
18	KPC-2	C2	8	>64	>64	>64	128	8	>8	256	1	32	2	256	4	8	32	>32	4	4	4	8	nt	>64	32	0.5	1
19	KPC-2	C2	2	>64	64	64	8	4	>8	8	1	4	1	8	2	16	16	>32	8	4	4	nt	>64	>64	8	0.25	8
20	KPC-2	C2	2	>64	64	32	8	2	>8	4	1	1	1	8	1	8	16	>32	8	4	4	4	>64	>64	16	0.25	>32
21	NDM-1	A	2	>64	>64	0.25	0.25	0.25	0.5	0.5	≤0.5	≤0.13	0.13	1	0.25	4	8	>32	1	0.5	0.5	4	>64	>64	>128	0.25	4
22	NDM-5	B	8	>64	64	64	32	2	2	256	1	16	0.5	256	0.5	8	16	>32	4	4	2	2	>64	>64	16	0.25	32
23	OXA-48	C2	>64	>64	64	64	>256	>32	>8	256	≤0.5	64	32	>256	>32	>16	>64	>32	16	0.13	32	4	>64	>64	64	0.25	8
24	OXA-48	C1	4	>64	64	16	>256	>32	>8	256	≤0.5	32	16	256	>32	16	>64	0.25	8	0.25	8	1	>64	>64	32	0.25	1
25	KPC-2	C2	2	>64	64	16	8	2	>8	1	≤0.5	2	0.5	4	1	8	16	>32	4	4	4	4	>64	>64	16	0.25	32
26	KPC-2	B	1	>64	64	32	4	2	0.13	1	≤0.5	2	0.25	2	1	8	32	0.25	4	2	1	2	>64	>64	16	0.25	1
27	KPC-2	A	8	>64	64	32	32	>32	8	64	1	2	2	256	>32	8	>64	16	16	0.25	2	2	>64	>64	>128	0.25	32
28	KPC-2	C2	2	>64	>64	>64	64	32	>8	16	≤0.5	16	16	32	16	16	16	>32	8	4	8	8	>64	>64	64	0.25	8

Supplementary Table S4. A summary of the most common AMR genes detected using the short read sequence data with 'genefinder', a PHE in-house mapping based gene detection program.

Antimicrobial Class	Gene	CP *E. coli*	CP *E. coli* (%)	Non-CP *E. coli*	Non-CP *E. coli* (%)
Beta-Lactamase	CTX-M-15	11	28.21%	61	32.45%
	OXA-9	10	25.64%	2	1.06%
	OXA-1	8	20.51%	50	26.60%
	TEM-191-p	7	17.95%	6	3.19%
	CTX-M-27	2	5.13%	3	1.60%
Aminoglycosides	aac(3)	15	38.46%	57	30.32%
	aac(6')	9	23.08%	55	29.26%
	aadA-5	14	35.90%	81	43.09%
	aph(6)-Id	15	38.46%	60	31.91%
	strB	15	38.46%	60	31.91%
	aadA-2	2	5.13%	7	3.72%
	aadA-22	2	5.13%	0	0.00%
	aadA-3	2	5.13%	0	0.00%
	aadA-8b	2	5.13%	8	4.26%
	aph(3')	3	7.69%	0	0.00%
Fluroquinolones	gyrA	28	71.79%	137	72.87%
	parC	25	64.10%	130	69.15%
Macrolides	ermB	2	5.13%	2	1.06%
	lnu-(F)	2	5.13%	0	0.00%
	mdf(A)	39	100.00%	186	98.94%
	mph-(A)	17	43.59%	83	44.15%
	strA	0	0.00%	64	34.04%
Trimethroprim	dfrA	25	64.10%	112	59.57%
Tetracycline	tet(A)	19	48.72%	113	60.11%
Sulfonamides	sul-1	20	51.28%	103	54.79%
	sul-2	14	35.90%	60	31.91%

Supplementary Table S5. A summary of the all plasmid associated origins of replication detecting using the short read sequence data with 'PHE-genefinder', an in-house gene detection program.

Metadata							FIA				FIB		
**Sequence ID**	**Clade**	**Carbapenemase**	**Transposon**	**Fim Type**	**CTX-M-15/27**	**A/C**	**FIA-1**	**FIA-2**	**FIA-4**	**FIA All**	**FIB-1**	**FIB-20**	**FIB-23**
1	A	KPC-2	unknown-1	fimH234	None	**0**	1	0	0	**1**	0	0	1
2	A	KPC-2	unknown-1	fimH41	None	**0**	1	0	0	**1**	1	0	0
3	A	KPC-2	unknown-1	fimH41	None	**0**	0	0	0	**0**	1	0	0
4	A	OXA-48	Tn1999	fimH41	None	**0**	1	0	0	**1**	0	0	1
5	A	OXA-48	Tn1999	fimH41	None	**0**	0	0	0	**0**	1	0	0
6	A	OXA-48	Tn1999-1	fimH41	None	**0**	0	0	0	**0**	1	0	0
7	A	OXA-48	Tn1999	fimH41	None	**0**	0	0	0	**0**	0	0	0
8	A	OXA-48	Tn1999	fimH41	None	**0**	0	0	0	**0**	0	0	0
9	A	VIM-1		fimH41	None	**1**	0	0	0	**0**	1	0	0
10	A	VIM-4		fimH41	None	**1**	0	0	0	**0**	1	0	0
11	B	KPC-2	Tn4401a-1	fimH22	CTX-M-15	**0**	0	0	0	**0**	0	0	0
12	B	KPC-2	Tn4401b-1	fimH22	None	**0**	0	0	0	**0**	0	0	0
13	B	KPC-2	Tn4401b-1	fimH22	None	**0**	0	0	0	**0**	0	0	0
14	B	KPC-2	Tn4401a-1	fimH376	None	**0**	0	0	0	**0**	1	0	0
15	B	NDM-1		Untypable	None	**0**	0	0	0	**0**	1	0	0
16	B	OXA-48	Tn1999	fimH22	None	**0**	0	0	0	**0**	1	0	0
17	C1	KPC-2	unknown-1	fimH30	None	**0**	0	1	0	**1**	0	1	0
18	C1	KPC-2	unknown-1	fimH30	None	**0**	0	1	0	**1**	0	1	0
19	C1	KPC-2	Tn4401a-1	fimH30	None	**0**	0	1	0	**1**	0	1	0
20	C1	KPC-2	Tn4401a-1	fimH30	None	**0**	0	1	0	**1**	0	1	0
21	C1	NDM-1		fimH30	CTX-M-15	**0**	0	1	0	**1**	0	1	0
22	C1	NDM-5		fimH30	CTX-M-15	**0**	0	1	0	**1**	0	1	0
23	C1	OXA-48	Tn1999	fimH30	None	**0**	0	1	0	**1**	0	1	0
24	C1	OXA-48	unknown-unknown	fimH30	CTX-M-27	**0**	0	1	0	**1**	0	1	0
25	C2	KPC-2	unknown-1	fimH30	CTX-M-15	**0**	1	0	0	**1**	0	1	0
26	C2	KPC-2	unknown-1	fimH30	None	**0**	0	0	0	**0**	0	0	0
27	C2	KPC-2	Tn4401a-1	fimH30	CTX-M-15	**0**	1	0	0	**1**	1	0	0
28	C2	KPC-2	Tn4401a-1	fimH30	None	**0**	1	0	0	**1**	1	0	0
29	C2	KPC-2	Tn4401a-1	fimH30	None	**0**	1	0	0	**1**	1	0	0
30	C2	KPC-2	Tn4401a-1	fimH30	None	**0**	1	0	0	**1**	1	0	0
31	C2	KPC-2	Tn4401a-1	fimH30	None	**0**	1	0	0	**1**	1	0	0
32	C2	KPC-2	Tn4401a-1	fimH30	None	**0**	1	0	0	**1**	1	0	0
33	C2	KPC-2	unknown-unknown	fimH30	CTX-M-15	**1**	1	0	0	**1**	1	0	0
34	C2	KPC-2	Tn4401a-1	fimH30	None	**0**	1	0	0	**1**	0	0	0
35	C2	NDM-1		fimH30	CTX-M-15	**1**	0	0	1	**1**	1	0	0
36	C2	NDM-1		fimH30	CTX-M-15	**0**	0	0	1	**1**	1	0	0
37	C2	OXA-181	unknown-unknown	fimH30	CTX-M-15	**0**	0	0	0	**0**	1	0	0
38	C2	OXA-48	Tn1999-1	fimH30	CTX-M-15	**0**	1	0	0	**1**	1	0	0
39	C2	OXA-48	Tn1999	fimH30	CTX-M-15	**0**	0	0	0	**0**	1	0	0

Sequence ID	FIB cont.		FII												L/M					
	FIB-24	FIB All	FII-1	FII-2	FII-4	FII-29	FII-31	FII-36	FII-45	FII-52	FII_71	FII-K2	FII All	I1	L/M-traU	L/M-parB	L/M All	N	P	Total
1	0	1	1	0	0	0	0	0	0	0	0	0	1	0	0	0	0	1	0	4
2	0	1	1	0	0	0	0	0	0	0	0	0	1	0	0	0	0	1	0	4
3	0	1	0	1	0	0	0	0	0	0	0	0	1	0	0	0	0	1	0	3
4	0	1	0	1	0	0	0	0	0	0	0	0	1	1	1	0	1	0	1	6
5	0	1	0	1	0	1	0	0	0	0	0	0	2	1	1	1	2	0	0	6
6	0	1	0	0	1	0	0	0	0	0	0	0	1	1	1	1	2	0	0	5
7	0	0	0	0	0	0	0	0	0	0	0	0	0	1	1	1	2	0	0	3
8	0	0	0	0	1	0	0	0	1	0	0	0	2	0	1	1	2	0	0	4
9	0	1	0	0	1	0	0	0	0	0	0	0	1	0	0	0	0	0	0	3
10	0	1	0	1	0	1	0	0	0	0	0	0	2	0	0	0	0	0	0	4
11	0	0	0	0	0	0	0	0	0	0	0	1	1	1	0	0	0	1	0	3
12	1	1	0	1	0	0	0	0	0	0	0	0	1	1	0	0	0	0	0	3
13	1	1	0	1	0	0	0	0	0	0	0	0	1	1	0	0	0	0	0	3
14	0	1	0	1	0	1	0	0	0	0	0	0	2	0	0	0	0	0	0	3
15	0	1	0	1	0	1	0	0	0	0	0	0	2	1	1	1	2	0	0	6
16	0	1	0	1	0	1	0	0	0	0	0	0	2	0	1	1	2	0	0	5
17	0	1	1	0	0	0	0	0	0	0	0	0	1	0	0	0	0	1	0	4
18	0	1	1	0	0	0	0	0	0	0	0	0	1	0	0	0	0	1	0	4
19	0	1	1	0	0	0	0	0	0	0	0	1	2	0	0	0	0	0	0	4
20	0	1	1	0	0	0	0	0	0	0	0	1	2	0	0	0	0	0	0	4
21	0	1	1	0	0	0	0	0	0	0	0	0	1	0	0	0	0	0	0	3
22	0	1	0	0	0	0	0	0	0	0	1	0	1	0	0	0	0	0	0	3
23	0	1	0	0	0	0	0	0	0	0	0	0	0	0	1	1	2	0	0	4
24	0	1	0	0	0	0	0	0	0	0	0	0	0	0	0	0	0	0	0	2
25	0	1	0	1	0	0	0	0	0	0	0	0	1	0	0	0	0	1	0	4
26	1	1	0	0	0	0	0	0	0	1	0	0	1	0	0	0	0	1	0	3
27	0	1	0	0	0	0	0	0	0	0	0	0	0	1	0	0	0	0	0	3
28	0	1	0	0	0	0	0	0	0	0	0	1	1	0	0	0	0	0	0	3
29	0	1	0	1	0	1	0	0	0	0	0	1	3	0	0	0	0	0	0	5
30	0	1	0	1	0	1	0	0	0	0	0	1	3	0	0	0	0	0	0	5
31	0	1	0	1	0	1	0	0	0	0	0	1	3	0	0	0	0	0	0	5
32	0	1	0	1	0	1	0	0	0	0	0	1	3	0	0	0	0	0	0	5
33	0	1	1	0	0	0	0	0	0	0	0	0	1	0	0	0	0	0	0	4
34	0	0	0	1	0	0	0	0	0	0	0	1	2	0	0	0	0	0	0	3
35	0	1	0	0	0	0	1	0	0	0	0	0	1	0	0	0	0	0	0	4
36	0	1	0	0	0	0	1	0	0	0	0	0	1	0	0	0	0	0	0	3
37	0	1	0	1	0	0	0	0	0	0	0	0	1	0	0	0	0	0	0	2
38	0	1	1	0	0	0	0	0	0	0	0	0	1	0	1	0	1	0	0	4
39	0	1	0	0	0	0	0	1	0	0	0	0	1	0	1	0	1	0	0	3
